# Supplementary material for: Coronavirus testing indicates transmission risk increases along wildlife supply chains for human consumption in Viet Nam, 2013-2014
Source: PLoS One. 2020 Aug 10;15(8):e0237129. doi: 10.1371/journal.pone.0237129 (PMC7416947; doi:10.1371/journal.pone.0237129)
Supplement: S1 Table — (PDF) [file pone.0237129.s001.pdf]

S1 Table. Summary of all testing results by genus, interface, sub-interface, sample types, sites, percentage of samples testing positive, and viral species.

Genus	Interface	Sub-interface	Sample type (sites)	% individual positive	Viral species (X|X indicates co-infection)
Cynopterus	Human dwelling	Natural bat roost	Oral swab (1)	0% (0/1)	
			Rectal swab (1)	0% (0/2)	
Pteropus	Human dwelling	Natural bat roost	Feces (1)	8.9% (4/45)	PREDICT_CoV-17, PREDICT_CoV-35
			Oral swab (1)	0% (0/13)	
			Rectal swab (1)	0% (0/15)	
			Urine (1)	0% (0/2)	
Micro-chiroptera[a]	Human dwelling	Bat guano farm	Feces (17)	76.5% (234/306)	Bat coronavirus 512/2005, PREDICT_CoV-35, PREDICT_CoV-35|Bat coronavirus 512/2005, PREDICT_CoV-17|Bat coronavirus 512/2005
		Bat guano farm	Urine (2)	0% (0/7)	
Field rats *(Rattus + Bandicota)*	Live rodent trade	Large market	Brain (5)	8.9% (4/45)	Murine coronavirus
			Feces (1)	0% (0/13)	
			Kidney (8)	4.9% (3/61)	Murine coronavirus
			Lung (3)	7.8% (4/51)	Murine coronavirus, Longquan aa coronavirus
			Oral swab (14)	32.1% (88/274)	Murine coronavirus, Longquan aa coronavirus, Murine coronavirus | Longquan aa coronavirus
			Rectal swab (1)	0% (0/1)	
			Small intestine (13)	22.6% (50/221)	Murine coronavirus, Longquan aa coronavirus,

					Murine coronavirus | Longquan aa coronavirus
			Urine swab (1)	0% (0/6)	
		Restaurant	Feces (1)	50.0% (1/2)	Longquan Aa mouse coronavirus
			Lung (2)	27.1% (23/85)	Murine coronavirus, Longquan aa coronavirus
			Oral swab (2)	51.3% (61/119)	Murine coronavirus, Longquan aa coronavirus
			Small intestine (2)	28.4% (27/95)	Murine coronavirus, Longquan aa coronavirus, Murine coronavirus | Longquan aa coronavirus
			Spleen (1)	50.0% (1/2)	Murine coronavirus
			Urine swab (1)	0% (0/4)	
		Trader	Brain (1)	4.0% (1/25)	Murine coronavirus
			Lung (4)	12.8% (6/47)	Murine coronavirus, Murine coronavirus | Longquan Aa mouse coronavirus
			Oral swab (8)	18.2% (28/154)	Murine coronavirus
			Small intestine (7)	12.1% (14/116)	Murine coronavirus, Longquan aa coronavirus
Rattus	Wildlife farm		Environmental sample (1)	100% (1/1)	Bat coronavirus 512/2005
Hystrix	Wildlife farm		Environmental sample (4)	7.1% (2/28)	Bat coronavirus 512/2005
			Feces (23)	6.0% (18/299)	Bat coronavirus 512/2005, Infectious bronchitis virus (IBV)
			Urine swab (3)	0% (0/4)	

Rhizomys	Wildlife farm		Feces (11)	6.3% (6/96)	Bat coronavirus 512/2005, Infectious bronchitis virus (IBV)
Sciuridae	Wildlife farm		Feces (1)	0% (0/1)	

[a] Suborder
