# Supplementary material for: Coronavirus testing indicates transmission risk increases along wildlife supply chains for human consumption in Viet Nam, 2013-2014
Source: PLoS One. 2020 Aug 10;15(8):e0237129. doi: 10.1371/journal.pone.0237129 (PMC7416947; doi:10.1371/journal.pone.0237129)
Supplement: S2 Table — (PDF) [file pone.0237129.s002.pdf]

S2 Table: Multivariate mixed effect logistic regression showing the association between season and sub-interface with coronavirus positives in field rats.

Variables	Categories	Odds Ratio	95% CI	P-value
Season	Dry	1.0		
	Wet	4.9	1.4 - 18.0	<0.01
Sub-interface	Rat trader	1.0		
	Large market	2.2	1.0 - 4.8	0.0326
	Restaurant	10.0	2.7 - 39.5	<0.01

Site was used as grouping variable (i.e. random effect).
