# Supplementary material for: Coronavirus testing indicates transmission risk increases along wildlife supply chains for human consumption in Viet Nam, 2013-2014
Source: PLoS One. 2020 Aug 10;15(8):e0237129. doi: 10.1371/journal.pone.0237129 (PMC7416947; doi:10.1371/journal.pone.0237129)
Supplement: S3 Table — (PDF) [file pone.0237129.s003.pdf]

	Sequence Name	Country	GenBank accession number
1	Bat/ VN13P0020/Vietnam/2013	Vietnam	KX285719
2	Coronavirus_PREDICT_CoV-17/PB015 = REFERENCE SEQ	Nepal	KX284941
3	Bat/VN13P0024/Vietnam/2013	Vietnam	KX285720
4	Bat/VN13F0063/Vietnam/2013	Vietnam	KX285605
5	Bat/VN13F0234/Vietnam/2013	Vietnam	KX285678
6	Bat/VN13F0226/Vietnam/2013	Vietnam	KX285672
7	Bat/VN13F0063/Vietnam/2013	Vietnam	KX285631
8	BtCoV/B551005/Pte_lyl/CB3-THA/Sep12	Thailand	MG256467
9	Coronavirus_PREDICT_CoV-35/KHP12PTR1-0160B	Cambodia	KX285751
10	Coronavirus_PREDICT_CoV-35/GVF-CM-ECO70504 = REFERENCE SEQ	Cameroon	KX284991
11	Coronavirus_PREDICT_CoV-35/CD115889 = REFERENCE SEQ	DRC	KX285074
12	Bat/VN13F0276/Vietnam/2013	Vietnam	KX285716
13	Bat/VN13F0161/Vietnam/2013	Vietnam	KX285642
14	Bat_coronavirus_BtCoV/A535/2005	China	DQ648824
15	Miniopterus_fuliginosus CYCU-M22/TW/2013	Taiwan	KT381920
16	Rhinolophus_monoceros_CYCU-R14/TW/2013	Taiwan	KT381916
17	Bat_coronavirus_HKU6_PREDICT-EHA-156-12-LS13721	China	KX285184
18	Porcine_epidemic_diarrhea_virus_isolate_KNU-1708	South Korea	MH052687
19	Human_coronavirus_229E	DRC	KX286258
20	NL63-related_bat_coronavirus_strain_BtKYNL63-9a	Kenya	NC_032107
21	Human_coronavirus_HKU1_isolate_SI17244	Thailand	MH940245
22	Rousettus_bat_coronavirus_HKU9_PREDICT-KHP13-BN1-0003	Cambodia	KX285758
23	Murine_coronavirus_KHP13-BLM2-0001 = REFERENCE SEQ	Cambodia	KX285756
24	Bovine_coronavirus_isolate_BCoV/SLO/5580/2013	Slovenia	KX059621
25	Betacoronavirus_HKU24_strain_HKU24-R05010I = REFERENCE SEQ	China	KM349744
26	Human_coronavirus_OC43_isolate_TNP_12643	Cote d'Ivoire	MG977452
27	Infectious_bronchitis_virus_strain_ck/CH/LHB/110825	China	KJ425488
28	Bat_coronavirus_isolate_BtCoV/B55080/S.hea/CB/Tha/1/2012	Thailand	KJ020603
29	SARS-related_coronavirus_isolate_F23	China	KU973688
30	Bat coronavirus HKU2 isolate HKU2-1	Hong Kong	DQ249235
31	Bat coronavirus HKU4 isolate HKU4-4	Hong Kong	DQ249216
32	Scotophilus bat coronavirus Bt/CoV/512/2005	China	DQ648858
33	Bat coronavirus HKU8 isolate HKU8-1	Hong Kong	DQ249228
34	Rousettus bat coronavirus HKU10 isolate 183A	China	JQ989270
35	Bat/VN13F0060/Vietnam/2013	Vietnam	KX285602
36	Bat/VN13F0065/Vietnam/2013	Vietnam	KX285606
37	Rat/VN13M0058/Vietnam/2013	Vietnam	MT221700
38	Rat/VN13M0002/Vietnam/2013	Vietnam	MT221698
39	Rat/VN13M0299/Vietnam/2013	Vietnam	MT221699
40	Rat/VN13M0005/Vietnam/2013	Vietnam	MT221701
41	Rat/VN14F0409/Vietnam/2014	Vietnam	MT221991
42	Rat/VN14F0491/Vietnam/2014	Vietnam	MT221995
43	Rat/VN14F0333/Vietnam/2013	Vietnam	MT221702
44	75-990L01-R5-CoV-BAT-VN	Vietnam	KX092205

45	75-55-L01-R4-CoV-BAT-VN	Vietnam	KX092189
46	75-98-L07-R4-CoVBAT-VN	Vietnam	KX092190
47	75-98-L07-R2-CoV-BAT-VN	Vietnam	KX092177
48	7562L01R6RATCoV	Vietnam	KX092217
49	7565L07R5RATCoV	Vietnam	KX092223
